# Advancements in one-dimensional protein structure prediction using machine learning and deep learning

**DOI:** 10.1016/j.csbj.2025.04.005

**Published:** 2025-04-03

**Authors:** Wafa Alanazi, Di Meng, Gianluca Pollastri

**Affiliations:** a School of Computer Science, University College Dublin, Belfield, Dublin D04 C1P1, Ireland; b Department of Computer Science, College of Science, Northern Border University, Arar, Saudi Arabia

**Keywords:** Deep learning, 1D protein prediction, Protein databases, Secondary structure, Intrinsic disorder, Solvent accessibility, AlphaFold, Protein language models

## Abstract

The accurate prediction of protein structures remains a cornerstone challenge in structural bioinformatics, essential for understanding the intricate relationship between protein sequence, structure, and function. Recent advancements in Machine Learning (ML) and Deep Learning (DL) have revolutionized this field, offering innovative approaches to tackle one- dimensional (1D) protein structure annotations, including secondary structure, solvent accessibility, and intrinsic disorder. This review highlights the evolution of predictive methodologies, from early machine learning models to sophisticated deep learning frameworks that integrate sequence embeddings and pretrained language models. Key advancements, such as AlphaFold’s transformative impact on structure prediction and the rise of protein language models (PLMs), have enabled unprecedented accuracy in capturing sequence-structure relationships. Furthermore, we explore the role of specialized datasets, benchmarking competitions, and multimodal integration in shaping state-of-the-art prediction models. By addressing challenges in data quality, scalability, interpretability, and task-specific optimization, this review underscores the transformative impact of ML, DL, and PLMs on 1D protein prediction while providing insights into emerging trends and future directions in this rapidly evolving field.

## 1 Introduction

Proteins are essential macromolecules, playing diverse roles across all forms of life [1]. From catalyzing biochemical reactions to mediating cellular signaling and facilitating molecular transport, their versatility stems from a remarkable structural complexity, organized hierarchically into primary, secondary, tertiary, and quaternary levels [2]. Predicting these structures, particularly from sequence data, is a cornerstone of structural bioinformatics, providing critical insights into the sequence-structure-function paradigm.

Despite the transformative impact of experimental techniques such as X-ray crystallography, nuclear magnetic resonance (NMR) spectroscopy, and cryo-electron microscopy, their limitations in scalability, cost, and technical feasibility leave a significant gap between known protein sequences and experimentally resolved structures [3], [4]. For instance, the Protein Data Bank (PDB) houses around 210,000 resolved structures, while UniProtKB contains over 250 million protein sequences [5]. Bridging this gap is crucial for advancing biomedical, biotechnological, and industrial applications.

Recent advances in computational biology, particularly machine learning (ML) and deep learning (DL), have revolutionized protein structure prediction. These methods automate feature extraction, model complex sequence-structure relationships, and analyze large-scale protein datasets with unprecedented accuracy. Unlike traditional approaches relying on curated features or homology-based templates, ML and DL frameworks directly utilize sequence data to predict essential properties, such as secondary structure, solvent accessibility, torsion angles, and intrinsic disorder.

One-dimensional (1D) protein annotations—including secondary structure, relative solvent accessibility, and intrinsic disorder—serve as foundational layers in the prediction of higher-order structures. These annotations not only improve understanding of protein folding and stability but also guide critical applications like protein-ligand interaction modeling, functional annotation, and rational protein design. The integration of natural language processing (NLP) methods, such as transformers and BERT-inspired architectures, has further advanced the field, enabling models to capture local and global dependencies within protein sequences and achieve superior performance even with limited labeled data [6].

Despite significant progress, protein prediction remains an evolving challenge. Key issues include the scarcity of high-quality training datasets, integrating multimodal data (e.g., sequence, structural, and functional), and developing scalable models capable of generalizing across diverse protein families. Moreover, the performance of existing models often varies significantly across tasks and datasets, emphasizing the need for standardized benchmarks and transparent evaluation methodologies.

The highlighted contributions of this review are enumerated as follows:

1. Comprehensive Exploration of Data Sources: The review provides an extensive ex- amination of general protein repositories such as UniProtKB and PDB, as well as specialized datasets for disordered proteins.
2. Advanced Sequence Representations**:** From basic one-hot encoding to advanced pre- trained language models (PLMs), the review delves into various representation techniques and their role in capturing sequence-specific and contextual information.
3. Methodological Insights: A detailed analysis of traditional ML methods (e.g., SVMs, Random Forests) and state-of-the-art DL architectures (e.g., CNNs, RNNs, transformers) is presented, highlighting their contributions to modeling protein complexity.
4. Core Prediction Tasks: The review emphasizes key tasks such as secondary structure prediction, solvent accessibility, and intrinsic disorder, alongside other structural features like torsion angles and contact density.
5. Evaluation Metrics and Benchmarks**:** A systematic discussion of evaluation metrics, including accuracy, F1 score, and Pearson correlation, is provided to guide model performance assessment and comparison.
6. Trends in 1D Protein Structure Prediction: The review introduces emerging technologies such as AlphaFold and protein language models (PLMs) while discussing their impacts, challenges, and future directions.
7. Challenges and Future Directions: The review identifies current limitations and suggests future research avenues, focusing on dataset diversity, multimodal integration, scalability, and interpretability. By synthesizing the latest advancements and challenges, this review aims to provide a nuanced understanding of the evolving landscape of 1D protein structure prediction and its implications for biological research and applications.

## 2 Data sources for 1D protein prediction

### 2.1 General protein databases

The Protein Data Bank (PDB) [7] is an indispensable repository in structural biology, housing experimentally determined three-dimensional structures of macromolecules,

including proteins and nucleic acids. Since its establishment in 1971, the PDB has evolved into a vast database with millions of entries, enabling atomic-level visualization of biomolecules. Although primarily focused on 3D structures, the PDB serves a critical role in 1D protein prediction tasks by linking protein sequences to structural annotations.

The PDB provides curated annotations such as amino acid sequences, secondary structure elements, and functional sites like binding regions. These annotations are leveraged by predictive tools like DSSP [8], [9], SCOP [10], [11], and MobiDB [12] to train models for tasks like secondary structure prediction, disordered region classification, and functional motif identification. Additionally, PDB data facilitates evolutionary analysis, creating conservation profiles for identifying functionally constrained regions, further enhancing 1D prediction tasks. Derived databases, including MobiDB and DisProt [13], integrate PDB-derived annotations to focus on IDRs, creating specialized resources for predictive modeling. By enabling high-quality dataset construction, the PDB remains a cornerstone for advancing computational biology.

UniProtKB [14] is a comprehensive and widely used database providing detailed information on protein sequences and functions. Organized into UniProtKB and UniRef, it supports diverse tasks, including protein annotation, classification, and structural prediction. UniProtKB is divided into Swiss-Prot, which provides manually curated, experimentally validated entries, and TrEMBL, which contains automatically annotated sequences. UniRef organizes sequences into clusters at varying identity thresholds (100 %, 90 %, and 50 %), reducing redundancy and optimizing alignment and functional annotation workflows. Databases like the Big Fantastic Database (BFD), containing billions of sequences, complement UniProt’s resources and facilitate protein structure prediction methods such as AlphaFold2.

The Database of Functional and Structural Annotations of Bacterial Proteins (DFB) is a specialized resource that provides curated functional and structural annotations exclusively for bacterial proteins, supporting research in bacterial genomics and protein characterization. The key distinction between DFB and UniProtKB is that DFB focuses solely on bacterial proteins with detailed functional and structural insights, while UniProtKB covers proteins from all domains of life, offering both manually curated and automatically annotated sequences for broader biological research. These datasets support advanced modeling of protein structure, function, and evolution across biological systems [15].Table 1 provides a summary of the primary datasets used in 1D protein prediction, highlighting their characteristics.

**Table 1:** Data Sources for 1D Protein Prediction.

**Resource/Dataset**	**Description**
UniProtKB [14]	Comprehensive protein sequence database with functional annotations.
PDB (Protein Data Bank) [7]	Repository of experimentally determined 3D protein structures.
MobiDB [12]	A resource combining experimental and computational annotations of IDRs for large-scale analyses.
DisProt [13]	Database of intrinsically disordered proteins (IDPs) and their structural properties.
PED (Protein Ensemble Database)[21]	Database of structural ensembles of IDRs, emphasizing their dynamic properties.
CASP (Critical Assessment of Structure Prediction) [22]	Benchmark competition evaluating protein structure prediction methods.
CAID (Critical Assessment of Intrinsic Disorder) [16]	A benchmarking initiative for IDR prediction tools, using high-quality datasets from DisProt and PDB.
CB513 [23]	A benchmark dataset of non-redundant, experimentally validated protein sequences for secondary structure prediction
TS115 [24]	A curated test set ensuring minimal sequence overlap for evaluating 1D structure predictors.

### 2.2 Specialized databases

The study of intrinsically disordered regions (IDRs) has been supported by specialized databases that curate and annotate IDR-related data. DisProt [13] and MobiDB [12] are key resources for studying IDRs. DisProt is a manually curated database that provides experimentally validated annotations for intrinsically disordered regions (IDRs) within proteins. Unlike computationally derived predictions, DisProt exclusively compiles disorder annotations from peer-reviewed literature, ensuring high-quality, evidence-backed data. These annotations serve as a gold standard for benchmarking IDR predictors and training machine learning models. However, since DisProt relies on experimental validation, it primarily covers well-studied proteins, limiting its application for large-scale or whole-protein analyses.

In contrast, MobiDB combines experimental and computational annotations, offering broader sequence coverage. Although it introduces variability due to predictive data, its comprehensive scope is valuable for large-scale analyses.

Both DisProt and MobiDB can be partially used for predictor evaluation, but their limitations must be carefully considered. For example, in the CAID [16], [17], [18] challenge, datasets for benchmarking IDR predictors were defined using DisProt annotations for disordered regions and PDB annotations for structured regions, while excluding regions without experimental data. This strategy ensures high-quality benchmark datasets for evaluating predictive tools.

Functional databases focus on specific roles of IDRs[19], [20]. PED (Protein Ensemble Database) [21] highlights structural ensembles of IDRs, emphasizing their dynamic properties. These specialized resources offer insights into IDR functionality and facilitate advancements in computational tools.

### 2.3 Testing datasets and benchmarks

The Critical Assessment of Protein Structure Prediction (CASP) [22] is a prestigious competition held every two years since 1994, where researchers and modelers are tasked with predicting the structure of previously unpublished proteins. The CASP test set is widely used in protein structure prediction, including 1D protein prediction tasks such as secondary structure, solvent accessibility, and other features. The inclusion of challenging targets, such as sequences with low homology to known structures and multi-domain proteins, makes the CASP test set an invaluable resource for advancing methods in 1D protein structure prediction.

The Critical Assessment of Intrinsic Disorder (CAID) initiative benchmarks computational tools for predicting IDRs in proteins. Like CASP, CAID fosters transparency and innovation through standardized evaluation frameworks. High-quality datasets derived primarily from DisProt and supplemented with PDB structural data serve as ground truth. Predictors are evaluated using metrics such as accuracy, precision, recall, F1 score, and Matthews correlation coefficient (MCC). CAID challenges include tasks like general IDR prediction, binding region prediction, and disordered flexible linker (DFL) prediction, driving innovation in IDR prediction methodologies.

Curated benchmark datasets such as CB513 [23], TS115 [24], TEST 2018 [25], and TEST 2020 [26] provide non-redundant, experimentally validated sequences for evaluation. These datasets, often derived from PDB and curated databases like ProteinNet and CullPDB, minimize sequence overlap with training data and ensure fair assessment of predictive models. By using well-established test sets or generating new ones, researchers evaluate how well 1D protein structure prediction models generalize across different properties such as SS, RSA, CN, and TA, ensuring predictive accuracy in real-world applications.

## 3 Methodologies for 1D protein prediction

Effective sequence preparation and feature extraction are critical steps in 1D protein structure prediction. These processes ensure that models are trained on diverse, representative, and non-redundant data while leveraging advanced embedding techniques to capture the biochemical, structural, and evolutionary features of protein sequences.

### 3.1 Sequence preparation

Reducing protein sequence redundancy is crucial in machine learning (ML) and deep learning (DL) tasks involving protein sequences, as it helps prevent overfitting, promotes

diverse representation of protein variants, and improves model generalization. Redundant sequences can distort results by artificially increasing dataset size without adding meaningful variation, which is particularly problematic in sequence-based prediction tasks where unique sequence patterns provide more valuable information than highly similar ones. By removing redundant sequences, models can focus on capturing distinct features, resulting in more accurate predictions and better computational efficiency.

Several tools are widely used for protein sequence redundancy reduction, including CD- HIT [27], which clusters similar sequences based on sequence identity to reduce redundancy; MMseqs2 [28], known for its fast clustering algorithms and ability to handle lower identity thresholds (down to 25 % sequence identity, compared to CD-HIT’s minimum of 40 %); and Clustal Omega [29], which aligns multiple sequences and aids in detecting and removing redundancy. These tools ensure that the data used for training ML and DL models is both diverse and representative of the full range of protein sequence variability.

### 3.2 Sequence representation

Protein sequence representation plays a critical role in computational biology, as protein sequences—composed of amino acids represented by letters—must be transformed into numerical forms for machine learning (ML) and deep learning (DL) analyses. Effective embedding techniques aim to capture key biochemical, structural, and evolutionary features in formats suitable for computational models.

One-Hot encoding [30] is a simple and widely used method for representing protein sequences. In this approach, each amino acid is represented as a binary vector, with one active dimension corresponding to the specific amino acid. Despite its simplicity, One-Hot encoding is valuable due to its universality and computational efficiency, making it a reliable baseline for protein sequence tasks. However, One-Hot encoding has limitations. It does not capture relationships between amino acids, such as biochemical properties or evolutionary context. As a result, while it retains raw sequence information, it lacks the depth needed for more complex tasks.

To overcome these limitations, One-Hot encoding is often combined with more advanced embedding techniques. When used alongside MSA-based embeddings or Protein Language Models (PLMs), it preserves sequence-specific details while benefiting from the richer, higher- level features provided by these methods. This combination helps mitigate overfitting, especially in small datasets, and enhances model performance by offering both raw sequence and abstracted contextual information.

Multiple Sequence Alignment (MSA) [31], [32] embeddings are among the most established approaches for protein sequence representation, leveraging evolutionary information encoded in conserved regions across homologous sequences. MSA captures biologically meaningful patterns, such as functional domains and structural motifs, which are critical for various prediction tasks.

Generating MSAs involves aligning a query sequence against a database of known protein sequences to identify homologous regions. Common tools used for this purpose include PSI- BLAST [33], a popular iterative sequence alignment algorithm; HHblits [34], which employs hidden Markov models for sensitive and rapid alignments; Clustal Omega [29], known for its accuracy in aligning diverse sequences; and MAFFT [35], recognized for its efficiency in handling large datasets. PSI-BLAST and HHblits are most commonly used, where HHblits is much faster than PSI-BLAST maintaining the similar searching ability, therefore HHblits is more popular in computing methods of studying protein sequences since computing methods often need to learn from a large amount of datasets [36], [37].

Once an MSA is generated, the alignment results are often transformed into statistical representations, such as amino acid frequencies at each sequence position, which can serve as input features for ML or DL models. Despite their effectiveness, MSA-based embed- dings face several challenges. These include reliance on homologous sequences—limiting their applicability for proteins with few or no known relatives, such as disordered regions [38]—and the computational cost of generating alignments, which can be resource-intensive and time- consuming. Moreover, MSA outputs often require additional processing to capture long- range dependencies, which may necessitate complex architectures like recurrent neural networks (RNNs) [39], [40] or hybrid models combining convolutional and recurrent layers [41]. In recent years, Protein Language Models (PLMs) have emerged as a transformative approach to protein sequence embedding, inspired by advances in natural language processing (NLP). Unlike MSA-based methods, PLMs operate directly on raw protein sequences, lever- aging large-scale training on protein databases such as UniProtKB [42] to learn representations that encode biochemical, structural, and evolutionary features.

Widely used PLMs include ProtTrans [43], a family of models pretrained on UniProtKB sequences that capture sequence-level relationships and contextual dependencies, and the Evolutionary Scale Modeling (ESM) [44], [45] models developed by Meta AI, which are particularly noted for their scalability and high-quality embeddings. TAPE [46] (Tasks Assessing Protein Embeddings) is another well-known framework, offering pretrained models and evaluation tasks for benchmarking embeddings. MSA Transformer [47] is a specialized PLM that combines MSA results with deep learning, allowing it to integrate evolutionary information alongside sequence-level features. Additionally, ProtGPT2 [48] has demonstrated the ability to design novel protein sequences, further showcasing the versatility of PLMs.

Compared to MSA-based methods, PLMs offer significant advantages. They do not de- pend on homologous sequences, making them faster and more applicable to proteins with limited evolutionary information. Furthermore, PLMs excel at capturing long-range dependencies and contextual relationships within sequences, producing embeddings that are Often more informative and easier to utilize than raw MSA outputs. However, open questions remain regarding the relative performance of PLMs and MSA-based methods for specific tasks, such as intrinsic disorder region prediction, and the choice of optimal embeddings often depends on the prediction task and dataset.

Recent efforts in protein sequence embedding increasingly focus on integrating the strengths of MSA-based methods and PLMs. This hybrid approach combines evolutionary signals from MSAs with the contextual richness of PLM-derived embeddings, aiming to improve predictive accuracy across a wide range of applications. While PLMs are rapidly gaining prominence, the computational biology community continues to explore their limitations and the potential for further refinement in specific prediction contexts.

### 3.3 Traditional machine learning approaches

Traditional machine learning (ML) methods have been widely employed in 1D protein sequence prediction, offering effective solutions in scenarios with limited data or computational resources. These approaches depend on carefully engineered features, such as evolutionary profiles, sequence composition, and physicochemical properties, to represent protein sequences numerically.

Machine learning became widely used in 1D protein sequence-based prediction in the 1990s and early 2000s, when researchers began applying computational models to classify protein structural and functional properties. Before this period, sequence-based predictions primarily relied on statistical methods and manual feature engineering, such as position-specific scoring matrices (PSSMs) and hidden Markov models (HMMs). As the field progressed, machine learning models such as support vector machines (SVMs) and random forests (RFs), gained popularity, offering more accurate predictions by incorporating sequence-derived features. These methods were used extensively in secondary structure prediction, intrinsic disorder prediction, and functional site identification. By the mid-2010s, ensemble ML models that combined multiple classifiers and evolutionary sequence information became the state-of-the-art approach for many prediction tasks.

Key ML methods include:

Support Vector Machines (SVMs): Utilize kernel functions to map data into higher- dimensional spaces, enabling the identification of complex relationships. SVMs have been particularly successful in predicting secondary structure and intrinsically disordered regions [49], [50].

Random Forests (RFs): Robust and interpretable models that aggregate decision trees to improve accuracy and prevent overfitting. They have been effectively applied to tasks such as solvent accessibility prediction and functional site identification [51].

k-Nearest Neighbors (k-NN): A similarity-based classification algorithm suited for residue-level predictions such as phosphorylation and glycosylation site prediction. It re- mains a reliable method for small datasets [52].

Ensemble Methods: Techniques like AdaBoost and gradient boosting combine multiple weak learners to improve performance. These methods are frequently employed in boundary prediction tasks and functional annotation of protein domains [53].

Traditional ML methods are valued for their computational efficiency and interpretability. However, they heavily depend on feature engineering and often face challenges in capturing long-range dependencies within protein sequences. While they remain particularly useful for tasks with limited annotated data or where interpretability is essential, the advent of Protein Language Models (PLMs), which offer richer representations of protein sequences, has significantly accelerated the shift towards deep learning methods, gradually reducing the reliance on traditional ML approaches.

### 3.4 Deep learning innovations

Deep learning (DL), a subset of machine learning, uses artificial neural networks with multiple layers to automatically learn hierarchical representations of data.The shift from machine learning to deep learning began in the mid-2010s[5], driven by greater computational power, larger sequence databases, and deep learning's success in other fields. Unlike ML, which required manual feature engineering, deep learning models automatically learned complex sequence patterns, leading to convolutional neural networks (CNNs), recurrent neural networks (RNNs), and transformer-based models outperforming earlier methods in protein sequence prediction[54].

Deep learning architectures differ from traditional machine learning (ML) models primarily in their depth—Deep learning architectures differ from traditional machine learning (ML) models primarily in their depth—the number of hidden layers in a neural network. While traditional ML includes models like Support Vector Machines (SVMs) and Random Forests, neural networks qualify as deep learning when they have multiple hidden layers (beyond a single-layer Perceptron).

The introduction of Multi-Layer Perceptrons (MLPs) marked the transition to DL, as these networks use layered architectures and backpropagation for training. The defining characteristic of DL is its ability to automatically extract hierarchical features from data, whereas traditional ML relies on manually engineered features. By leveraging large datasets and high computational power, deep learning has achieved state-of-the-art performance in diverse fields such as image recognition, natural language processing, and bioinformatics. Its key advantage lies in its ability to extract meaningful features directly from raw data, eliminating the need for extensive manual feature engineering. With architectures like convolutional neural networks (CNNs), recurrent neural networks (RNNs), and transformers, deep learning models excel at capturing complex patterns and long-range dependencies. These methods have become particularly transformative in analyzing biological sequences, enabling breakthroughs in protein structure prediction and functional annotation [54], [55], [56]. These methods have set benchmarks in tasks such as secondary structure prediction, disorder region identification, and functional site annotation. Below, we discuss the key deep learning architectures applied in this domain.

Recurrent Neural Networks (RNNs): Tailored for sequential data processing, RNNs [39] and bidirectional RNNs (BRNNs) [57] process sequences in both forward and reverse directions, making them suitable for protein sequence prediction tasks [58]. Their ability to maintain a hidden state that evolves with the sequence allows modeling of dependencies within the sequence. However, the vanishing gradient problem often limits their capacity to capture long-range dependencies effectively.

Long Short-Term Memory Networks (LSTMs): Address the limitations of RNNs by employing gating mechanisms, which regulate the flow of information and mitigate vanishing gradients. Bidirectional LSTMs (BiLSTMs) [59], an extension of this architecture, have been particularly effective in tasks like intrinsic disorder prediction and secondary structure classification.

Convolutional Neural Networks (CNNs): Highly effective at detecting spatially localized patterns in protein sequences, CNNs [55], [60] are particularly strong in tasks like residue- level annotations and secondary structure prediction, where hierarchical feature extraction allows for identifying patterns across multiple scales.

Convolutional and Bidirectional Recurrent CNN (CBRCNN)**:** Combines CNNs for local pattern detection with bidirectional recurrent layers (e.g., BiLSTMs or BRNNs) for capturing sequential relationships. This hybrid architecture is especially advantageous for tasks such as solvent accessibility prediction, where both spatial and sequential features are critical [41].

Transformers: Transformers [61], [62] have recently emerged as a dominant architecture for protein sequence prediction, owing to their ability to capture long-range dependencies and global context more effectively than traditional models like RNNs and CNNs. Pretrained Transformer models, such as ESM [44], [45] and ProtTrans [43], utilize transfer learning to generate high-quality embeddings, significantly enhancing the performance of various protein prediction tasks. These embeddings have proven particularly useful in tasks like secondary structure and disorder prediction [63].

Deep learning methods have become the cornerstone of protein sequence prediction, offering unparalleled performance and flexibility. The selection of an appropriate architecture depends on the task requirements, dataset characteristics, and computational resources, with each method offering unique advantages for specific applications.

During training, machine learning models must balance bias and variance to avoid underfitting or overfitting. Traditional ML models, with fewer parameters, are less prone to overfitting but may underfit if overly simplistic. Deep learning models, in contrast, have high capacity and are more susceptible to overfitting due to their complexity.

To manage this, datasets are typically split into training, validation, and test sets. The validation set helps tune hyperparameters and determine when to stop training. Overfitting is a frequent issue in deep learning, as large models can capture noise rather than meaningful patterns. Strategies such as regularization, early stopping, and data augmentation help mitigate this. Conversely, underfitting occurs when a model is too simple to learn the data’s complexity and can be addressed by increasing model capacity, improving feature representations, or extending training.

Striking the right balance is essential, particularly in sequence-based methods, where deep networks must generalize effectively without becoming overly complex.

## 4 Core 1D protein prediction tasks

**Fig. 1:**
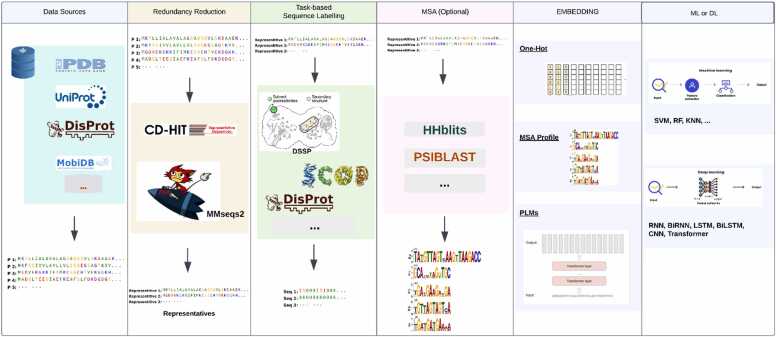
An Overview of the Workflow and Methodologies Used in 1D Protein Structure Prediction Note: MSA (Multiple Sequence Alignment) is optional due to the availability of pre- trained language models (PLMs). Evaluation methods are not shown in the diagram, as we consider them part of the ML or neural network training process.

One-dimensional (1D) protein structure prediction tasks simplify three-dimensional (3D) protein structures into linear features such as secondary structure (SS), relative solvent accessibility (RSA), and intrinsically disordered regions (IDR). Additional descriptors, like contact number (CN) and torsion angles (TA), further enrich our understanding of protein properties. These 1D features form the foundation for higher-order structure predictions, facilitating advancements in computational biology. This section explores established tasks, starting with secondary structure prediction, and examines emerging features and their predictors (Table 2), referencing shared architectures and strategies (Fig. 1)

**Table 2:** Deep Learning Methods for 1D Protein Structural Prediction.

**Year**	**Predictor**	**Task**	**Input Features/Encoding**	**DL Method/Architecture**
2005	Porter [68]	SS	PSSM (PSI-BLAST profiles)	Ensemble of Bidirectional RNNs
2006	Spritz [91]	IDR general	One-hot + MSA	SVM
2008	ANGLOR [115]	TA	Sequence profiles, predicted secondary structure, solvent accessibility	Composite Machine Learning (NN + SVM)
2012	ESpritz [92]	IDR general	One-hot + MSA	BRNN
2013	PaleAle 4.0 [89]	RSA	PSI-BLAST frequency profiles	Cascaded Bidirectional Recurrent Neural Networks
2014	SSpro/ACCpro 5 [86]	RSA, SS	PSI-BLAST profiles, sequence-based structural similarity	Ensemble of BRNNs combined with sequence-based structural similarity
2015	AcconPred[80]	RSA, CN	Evolutionary, structural, and amino acid features	Multitask Learning with Conditional Neural Fields
2016	RaptorX-Property [88]	RSA, SS, DISO	PSI-BLAST sequence profiles	Deep Convolutional Neural Fields (Deep-CNF) capturing sequence-structure relationships
2017	BMRSA[87]	RSA	PSSM, HMM profiles, and physicochemical properties	Deep learning framework with hybrid convolutional and recurrent network
2017	SPIDER2[69]	RSA, ASA, SS, TA, CN, HSE	PSI-BLAST and HHblits profiles, physicochemical properties	LSTM-BRNN networks capturing short- and long-range dependencies
2017	IUpred [93]	IDR general	None	Energy estimation
2017	MobiDB-lite [96]	IDR general	–	Ensemble
2018	SPIDER3[73]	RSA, ASA, SS, TA	One-hot encoding	LSTM-BRNN
2018	SPOT-disorder[99]	IDR general	MSA + physicochemical properties	LSTM + ResNet
2019	SPOT−1D[79]	RSA, ASA, SS, TA, CN, HSE	PSI-BLAST and HHblits profiles, physicochemical properties, predicted residue-residue contact maps	Ensemble of LSTM-BRNN and ResNet models integrating short- and long-term dependencies
2019	NetSurfP−2.0 [15]	RSA, ASA, SS, TA, disorder	One-hot encoding and HH-suite or MMseqs2 sequence profiles	CNNs + BiLSTM
2019	PaleAle 5.0 [81]	RSA	Clipped evolutionary profiles (PSI-BLAST, HHblits)	Stacked BRNN-CNN for long-range context extraction and refinement
2019	SPOT-disorder2[99]	IDR general	MSA + physicochemical properties	LSTM + ResNet
2020	Brewery [113]	SS, RSA, CD, and Structural Motifs	Evolutionary information from UniProt20, UniRef90, and BFD	Cascaded Bidirectional Recurrent and Convolutional Neural Networks
2020	OPUS-TASS[116]	SS, TA	Sequence, structural profiles	Ensemble Neural Networks
2021	DNSS2 [64]	SS	PSSM, HMM profiles, Atchley factors, MSA probabilities	Ensemble of CNN, RCNN, ResNet, CRMN, FractalNet, and InceptionNet
2021	DMVFL-RSA [82]	RSA	PSSM, PSFM, predicted secondary structure (PSS), three-state RSA probabilities (RPRSA)	Deep Multi-View Feature Learning (DMVFL) combining BiLSTM, SENet, and fully connected layers
2021	IUpred3 [94]	IDR general	None	Energy estimation
2021	MobiDB-lite 3.0 [97]	IDR general	–	Ensemble
2022	SPOT−1D-LM [75]	RSA, ASA, SS, TA, CN, HSE	One-hot encoding, ProtTrans and ESM−1b embeddings combined	Ensemble of ResNet and BiLSTM models capturing short- and long-term dependencies
2022	SSpro/ACCpro 6 [81]	RSA, SS	PSI-BLAST-derived sequence profiles and structural similarity	Ensemble of biGRU with convolutional and fully connected layers
2022	NetSurfP−3.0 [74]	RSA, ASA, SS, TA, disorder prediction	ESM−1b embeddings (1280 features per residue)	1D CNNs + BiLSTM
2023	IGPRED-MultiTask [83]	SS, TA, RSA	Sequence profiles, structural profiles, physicochemical properties	CNN, GCN, BiLSTM with Multi-Task Learning
2023	AttSec [77]	SS	ProtT5 embeddings	Transformer
2024	MFTrans[78]	SS	Hybrid (evolutionary profiles + embeddings)	CNN + BiGRU + Transformer
2024	AIUpred [95]	IDR general	Integer-based tokenization	Transformer
2024	PUNCH2 [100]	IDR general	One-hot + MSA-transformer + ProtTrans	CNN
2024	PUNCH2-light	IDR general	One-hot + ProtTrans	CNN
2025	Porter 6 [76]	SS	ESM2	CBRNN
2025	PaleAle 6 [90]	RSA	ESM2	CBRNN

This table presents deep learning-based methods for 1D PSA prediction, highlighting the models adopted and data encoding techniques used.

### 4.1 Protein secondary structure prediction (PSSP)

Protein secondary structure prediction is a fundamental component of structure prediction pipelines. Acting as a critical intermediary step, PSSP enables accurate modeling of tertiary structures and reveals important structural motifs. Secondary structures primarily include three-state and eight-state predictions. Three-state prediction simplifies classifications into helix (H), strand (E), and coil (C). Eight-state prediction, a finer categorization introduced by Kabsch and Sander, expands to eight structural elements, including motifs like *π*-helices and isolated *β*-bridges, among others.

The DSSP program remains the gold standard for assigning secondary structures in the Protein Data Bank (PDB). Its 2021 update (DSSP 4.0) added the poly-proline helix (P), extending the original eight-class scheme. This nine-class system was applied in recent studies leveraging lightweight fine-tuning of pretrained models [64], showcasing evolving approaches to PSSP.

#### 4.1.1 PSSP predictors

Advances in PSSP reflect a journey from traditional machine learning (ML) to modern deep learning (DL) architectures, optimizing input features and computational strategies. Early models utilized Support Vector Machines (SVMs) [65] and Hidden Markov Models (HMMs) [66]. For instance, HMMSTR demonstrated the utility of sequence-structure motifs, while Yuan [67] used SVMs with RBF kernels for contact number predictions.

Bidirectional Recurrent Neural Networks (BRNNs) [68] marked a significant shift, capturing forward and backward contextual information. Ensemble BRNNs with shortcut connections, such as Porter [68], refined these methods for enhanced accuracy. Convolutional Neural Networks (CNNs) and Long Short-Term Memory (LSTM) networks revolutionized PSSP. Models like SPIDER2 [69] captured both local and non-local dependencies, while DeepACLSTM [70] combined asymmetric CNNs with bidirectional LSTMs. Porter 5 [71] employed ensembles of models to mine evolutionary information effectively.

Addressing the challenges of evolutionary profiles, single-sequence predictors like SPOT- 1D-Single [72] andSPIDER3-Single [73]bypassed sequence alignments by utilizing one-hot encoded inputs, offering solutions for proteins with few homologs. Language models such as ESM-1b [77] and ProtT5 [43] have transformed PSSP by providing sequence embeddings that encode structural and contextual information without requiring alignments. Predictors like NetSurfP-3.0 [74], SPOT-1D-LM [75], and Porter 6 [76] leverage pretrained protein language model (PLM) embeddings, such as ESM-1b,ESM 2 and ProtTrans, allowing them to efficiently model sequence relationships without relying on multiple sequence alignments (MSAs). These models employ deep learning architectures, including convolutional neural networks (CNNs), bidirectional LSTMs (BiLSTMs), and hybrid recurrent-convolutional networks (CBRCNNs), enabling them to capture both local and long-range dependencies in protein sequences. SPOT-1D-LM integrates embeddings from ESM-1b and ProtTrans to improve the accuracy of 1D structural predictions, balancing computational efficiency with high predictive performance. Porter 6 optimizes sequence representation using a CBRCNN framework, while NetSurfP-3.0 streamlines feature extraction with CNN-BiLSTM models trained on PLM embeddings. By eliminating the need for evolutionary profiles and leveraging self-supervised learning, these models significantly enhance scalability, generalization, and prediction speed, making them particularly useful for studying proteins with limited evolutionary information.

Advanced architectures like AttSec [77] employ transformer embeddings (e.g., ProtT5) to capture long-range dependencies. Hybrid models, such as MFTrans [78], combine CNNs, BiGRUs, and transformers for comprehensive feature extraction. Ensemble methods, including SPOT-1D [79], SPOT-1D-LM [75] and DNSS2 [64], further enhance performance through integration of diverse models. The evolution of PSSP—from traditional ML techniques to cutting-edge transformer models—highlights a relentless drive for precision, efficiency, and scalability in protein structure prediction.

### 4.2 Solvent accessibility (RSA)

The concept of protein solvent accessibility, introduced by Lee and Richards in 1971 [80], remains foundational in understanding the spatial arrangement of residues during protein folding. Solvent accessibility, defined as the surface area of a protein residue accessible to a solvent, provides critical insights into the hydration properties and solvent environment of amino acids within a protein [81]. This measure has been extensively used to analyze protein structure and function, serving as an essential intermediate feature in studies of protein folding [82].

Solvent accessibility plays a critical role in protein folding, as it reflects the spatial arrangement and packing of residues. This arrangement is largely driven by the hydrophobic effect, where hydrophobic residues are buried within the protein core while hydrophilic residues remain exposed to the solvent. This principle not only drives folding in monomeric soluble globular proteins but also highlights the intricate relationship between protein structure and function [80].

In addition to its structural role, solvent accessibility is a key factor in immunogenicity prediction, as surface-exposed residues frequently serve as antigenic sites. This is particularly relevant for vaccine design, antibody engineering, and cross-reactivity assessment, where solvent-accessible regions influence immune recognition. While immunogenicity prediction extends beyond 1D structure prediction, solvent accessibility remains a key feature in evaluating immune interactions.

Relative solvent accessibility (RSA) is a normalized measure of a residue’s solvent- ex- posed surface area, calculated as a percentage of the maximum possible exposure for that amino acid type. RSA prediction is often the first step in characterizing protein structure and function. While traditionally treated as a multi-class classification problem, RSA pre- diction is frequently simplified to a binary classification task using a 25 % threshold, where residues with RSA below this value are categorized as "buried," and those above it as "ex- posed." This classification system has been further refined into two-class, three-class, or four-class predictions, as well as continuous real-valued predictions [82].

The calculation of RSA relies on absolute solvent accessibility (ASA) values derived from experimentally resolved 3D structures processed by tools such as DSSP [8], [9]. These ASA values are normalized using the formula:

$$\mathrm{RS}A_{i}=\frac{SA_{i}}{\mathrm{MA}X_{i}}\;*\;100\%$$

where *SAi* is the solvent accessibility of residue i(in Å²) from DSSP, and *MAXi* is the maximum solvent accessibility for that amino acid i (in Å²) type.

In addition to its biological importance, solvent accessibility significantly aids protein structure prediction. Unlike complex global features like contact maps or distance matrices, RSA is easier to compute and predict, making it a valuable component in many protein prediction pipelines. RSA also complements local 1D features such as secondary structure and backbone torsion angles, effectively bridging the gap between 1D and 3D structural information [83].

#### 4.2.1 RSA predictors

The prediction of RSA has evolved significantly, transitioning from traditional machine learning approaches to state-of-the-art deep learning models. Early methods relied on multi- class or binary classification frameworks using machine learning algorithms such as neural network-based regression, k-nearest neighbor [84], and support vector machines [85]. These methods achieved moderate accuracy, but their reliance on handcrafted features limited their scalability.

Discrete-valued predictors like SSpro/ACCpro 5 [86], SSpro/ACCpro 6 [81], BMRSA [87], RaptorX-Property [88], PaleAle 4.0 [89], and PaleAle 5.0 [90] were instrumental in advancing RSA prediction. SSpro/ACCpro 6 introduced significant advancements in accuracy through enhanced input features (e.g., 232 features per residue position) and structural information from homologous segments in the Protein Data Bank (PDB). It achieved an accuracy of 81.06 % for RSA prediction at a 25 % threshold on redundancy-reduced test sets from 2019, surpassing its predecessor SSpro/ACCpro 5 by 3–4 % in Q3 accuracy.

PaleAle 5.0, for instance, employed a stacked BRNN-CNN architecture, achieving accuracies of 80.5 %, 66.4 %, and 56.5 % for two-class, three-class, and four-class RSA predictions, respectively. These models utilized innovative features like "clipped" evolutionary profiles to enhance performance. Similarly, BMRSA achieved an 86.3 % accuracy for binary RSA classification by integrating position-specific scoring matrices, secondary structure, and a novel buried-exposed profile. RaptorX-Property used a Deep Convolutional Neural Fields (DeepCNF) model to achieve a Q3 accuracy of 66.3 % on CASP targets, capturing complex sequence-structure relationships without relying on structural templates.

Real-valued RSA predictors have addressed the limitations of discrete-valued approaches by providing continuous values for solvent accessibility. Examples include SPIDER2, SPIDER3-Single, NetSurfP-2.0 [15], SPOT-1D [79], and PaleAle 6. These predictors leverage deep learning techniques to extract complex sequence-structure relationships, significantly advancing RSA prediction accuracy.

Recent advancements have introduced deep multi-view learning frameworks like DMVFL- RSA [82], which combines BiLSTM, squeeze-and-excitation networks (SENet), and fully connected layers. DMVFL-RSA achieved an 81.0 % accuracy for 2-state RSA prediction and a PCC of 0.71 for real-valued RSA on the TEST524 dataset.

NetSurfP-3.0, SPOT-1D-LM, and PaleAle 6 leverage protein language model (PLM) embeddings to bypass the need for computationally expensive sequence alignments in predicting relative solvent accessibility (RSA). These predictors showcase comparable or superior performance to traditional profile-based methods, underscoring the transformative impact of PLMs on RSA prediction. Specifically, NetSurfP-3.0 achieved a Pearson correlation co- efficient (PCC) of 0.793 for RSA prediction on the CB513 dataset, while SPOT-1D-LM demonstrated robust performance with a PCC of 0.731 on the TEST2020 dataset. Similarly,

PaleAle 6 achieved a PCC of 0.7788 on the 2022 Test Set and 0.7308 on the more challenging 2024 Test Set, highlighting its strong performance and robustness in real-value RSA predictions.

### 4.3 Intrinsic disorder prediction (IDR)

Intrinsically disordered regions (IDRs) are protein segments that lack a fixed three- dimensional structure under physiological conditions. Their structural flexibility enables them to participate in diverse biological processes, such as molecular recognition, signaling, and regulation. Predicting IDRs and their specific functional regions is a key challenge in computational biology, with implications for understanding protein function and its association with diseases. IDRs can broadly function as binding regions, which interact with molecular partners, or non-binding regions, which perform functions independently of direct interactions. Among non-binding regions, disordered flexible linkers (DFLs) are especially prominent; over 75 % of non-binding IDRs are classified as DFLs, according to the DisProt database. As a result, this section gives special attention to DFL and binding region prediction tools.

IDR prediction methods can be broadly categorized into general IDR prediction, which classifies each residue in a sequence as ordered (0) or disordered (1), and prediction of specific functional regions within IDRs, such as DFLs and binding regions. These functional IDR predictors focus on regions involved in particular biological roles, with DFLs and binding regions being the most extensively studied categories.

#### 4.3.1 General IDR predictors

General IDR prediction involves the binary classification of protein residues as either ordered or disordered. This task has seen significant advancements over the years, with predictors evolving from traditional machine learning methods to more sophisticated deep learning models.

Spritz [91] was among the earliest predictors, utilizing support vector machines (SVMs) to classify residues based on sequence-derived features such as amino acid composition and physicochemical properties. It was trained on experimentally validated data from DisProt and PDB, providing reasonable accuracy for its time. However, Spritz’s reliance on SVMs limited its ability to capture long-range dependencies within sequences. Building on this foundation, ESpritz [92] introduced Bidirectional Recurrent Neural Networks (BRNNs) [57], which process sequence context in both forward and reverse directions. This approach significantly enhanced sensitivity and accuracy, particularly for longer disordered regions. Trained on a combination of PDB and DisProt-derived annotations, ESpritz remains competitive and is actively used in platforms like MobiDB.

IUPred [93] introduced a distinct, unsupervised methodology by predicting disorder based on residue-residue interaction energies. Its energy potential calculations efficiently identify regions unlikely to form stable tertiary structures. While its simplicity ensures speed and wide applicability, it sometimes lacks the precision of supervised models. IUPred3 [94] improved upon this by integrating evolutionary conservation, leveraging sequence alignments to refine energy estimations. This innovation enhanced precision and recall, particularly for conserved disordered regions. A further evolution, AIUPred [95], combines traditional energy-based calculations with deep learning techniques. By training a neural network on

DisProt, PDB, and MobiDB-derived annotations, AIUPred captures both global disorder trends and local residue-specific nuances. Its hybrid approach achieves superior performance, ranking 14th in CAID challenge round 3 on the Disorder PDB benchmark set.

Consensus-based methods, such as MobiDB-lite [96] and its successor MobiDB-lite 3.0 [97], aggregate predictions from multiple tools. These methods are particularly effective for short disordered regions and offer fast, specific predictions. Built on outputs from predictors trained on DisProt and PDB, MobiDB-lite 3.0 refines the consensus approach with improved speed and accuracy.

Deep learning advancements are exemplified by the SPOT-Disorder series. SPOT- Dis- order [98] integrates recurrent neural networks (RNNs) and convolutional neural net- works (CNNs) to capture both local and global sequence patterns. Using training data from Dis- Prot, PDB, and evolutionary alignments, it delivers high accuracy. SPOT-Disorder2 [99] further enhances this framework with attention mechanisms and advanced sequence embed- dings, achieving top-tier performance in metrics like accuracy and Matthews Correlation Coefficient (MCC). It consistently ranks in the top five predictors in CAID challenges.

PUNCH2 [100], [101] represents a novel approach, addressing challenges such as limited training datasets and effective feature extraction. It uses protein sequences as input with- out relying on additional features. Trained on sequences from experimental PDB datasets and fully disordered DisProt annotations, PUNCH2 employs ProtTrans-based embeddings for superior accuracy. Its architecture, featuring multiple convolutional layers, balances predictive confidence and computational efficiency. PUNCH2 and its streamlined version, PUNCH2-light, demonstrated their effectiveness by outperforming competitors in the CAID challenge round 3, securing first place [16], [18].

Overall, the progression from early methods like Spritz and IUPred to advanced predictors such as PUNCH2 and SPOT-Disorder2 highlights the field’s evolution. Traditional models provided a foundation of efficiency and interpretability, while modern deep learning and hybrid approaches deliver unparalleled accuracy and versatility. This comprehensive toolbox now supports diverse applications, from rapid analyses to in-depth investigations of complex protein sequences.

#### 4.3.2 DFL and binding region predictors

The prediction of disordered flexible linkers (DFLs) and binding regions within intrinsically disordered regions (IDRs) is an important yet specialized task in protein sequence analysis. These regions play crucial roles in protein flexibility, molecular recognition, and interaction. While general IDR prediction tools have made significant strides, DFL and binding region prediction remain challenging areas with fewer available tools. However, recent advancements have significantly improved prediction accuracy and applicability.

DFL Prediction: Disordered Flexible Linkers (DFLs) are non-binding regions within IDRs that provide structural flexibility and are essential for protein dynamics and function. Accurately identifying these regions is vital for understanding protein motion and its inter- action mechanisms. Several tools have been developed to predict DFLs, each contributing unique approaches to enhance accuracy and reliability.

DFLpred [102] is one of the pioneering tools for predicting DFL regions. Using machine learning techniques, DFLpred classifies residues based on sequence-derived features, achieving high-throughput predictions. Trained on experimentally validated datasets from DisProt and PDB, DFLpred provides reasonable accuracy for identifying DFLs. However, its reliance on manually crafted features limits its adaptability to novel datasets, which constrains its ability to generalize across diverse proteins.

APOD [103] improves upon DFLpred by incorporating advanced sequence-based features and refined model architectures. It achieves higher precision and recall, especially for short and ambiguous linker regions, providing a more robust tool for functional annotation. APOD’s ability to predict complex linker regions with higher accuracy has made it an important advancement in the field.

TransDFL [104] introduces transfer learning into DFL prediction. By pretraining on large protein sequence datasets and fine-tuning on specific DFL annotations, TransDFL significantly enhances prediction accuracy. This approach allows the model to generalize better across various protein families, making it a valuable tool for predicting DFLs in diverse datasets.

DisoFLAG [105] represents the latest innovation in DFL prediction. It utilizes a graph- based protein interaction model combined with a protein language model, which allows it to capture both local and global interaction patterns within protein sequences. This approach results in unprecedented accuracy for DFL prediction and demonstrates robustness across various datasets, establishing DisoFLAG as a state-of-the-art tool in the field.

Binding Region Prediction: Binding regions within IDRs are crucial for mediating protein-protein and protein-ligand interactions, playing a vital role in protein function and cellular processes. Accurate identification of these binding regions is essential for under- standing protein interaction networks and their functional implications.

ANCHOR2 [106], an extension of the original ANCHOR algorithm [107], predicts binding regions within disordered proteins. ANCHOR2 identifies segments within IDRs that cannot independently form stable structures but are capable of disorder-to-order transitions upon binding. Integrated into the IUPred2A web server, ANCHOR2 allows users to simultaneously predict disordered regions and their potential binding sites, offering an effective tool for binding site identification.

Ligand binding can induce structural changes in proteins, often stabilizing or reshaping secondary structure elements. While many cases involve disorder-to-order transitions, where intrinsically disordered regions (IDRs) fold upon ligand interaction, other structural modifications also occur. For example, some helices convert to sheets, and certain β-sheet regions undergo local unfolding and refolding to accommodate ligands. These dynamic changes present challenges for 1D structure prediction, as models must account for sequence-dependent flexibility and functional adaptability.

IDBindT5 [108] leverages advanced protein embeddings and machine learning techniques to predict binding residues within IDRs. By utilizing sequence information and embedding methods, IDBindT5 accurately identifies residues that are involved in binding, aiding in the functional annotation of disordered proteins. Its deep learning-based approach improves prediction accuracy, particularly for complex binding sites in disordered regions.

DEPICTER [109], which stands for DisorderEd PredictIon CenTER, is a comprehensive web server designed for predicting not only intrinsically disordered regions but also their associated molecular functions and binding partners. DEPICTER includes a state-of-the- art disorder predictor, flDPnn [110], and several other modern methods to predict different functions associated with disordered regions, providing a holistic approach to understanding protein disorder and its implications in molecular interactions.

These tools represent a significant leap forward in the computational prediction of binding regions within IDRs. By employing innovative methodologies such as machine learning, sequence embeddings, and disorder-to-order transition models, these predictors provide accurate and reliable identification of binding.

### 4.4 Additional structural features

#### 4.4.1 Contact number and contact density

Contact Number and Contact Density are 1D features that capture essential aspects of 3D protein structure, acting as simplified representations of contact maps. These features are inherently derived from the folding process and the spatial organization of protein residues[80].

The Contact Number, also referred to as the coordination number, is defined as the number of neighbouring residues within a specified radius, often measured from the C*β* atoms (or the C*α* atoms if C*β* is absent)[111].The contact number of a residue is an essential descriptor of the folding outcome, encapsulating the local structural environment. It has been suggested that, given the contact number for each residue, the possible protein conformations satisfying the contact number constraints are highly restricted[111]. As a result, predicted contact numbers serve as valuable constraints for de novo structure prediction and as effective inputs for contact map prediction[80] and useful for torsion angle prediction[111].

These features bridge the gap between protein sequence and structure, highlighting their importance in understanding and modelling protein conformations[80].

Contact Density (CD), sometimes referred to as contact occupancy or normalized contact number, builds on the concept of CN by normalizing it relative to the expected number of contacts or deriving it from eigenvectors of contact maps. This metric highlights the relative spatial packing density of residues, making it particularly useful for folding constraint analysis[112]. Brewery[113], a deep learning-based tool, extends the utility of CD by predicting it into four states: very low density, low density, high density, and very high density. This classification provides a more granular understanding of residue packing levels and simplifies the analysis of spatial density distributions in proteins.

Both CN and CD are essential for protein structure prediction because they simplify complex 3D spatial information into manageable 1D features. By representing the spatial packing constraints that arise from protein folding, these features allow for more efficient prediction and analysis of protein conformations. Furthermore, their ability to encode structural information in a compact form makes them a cornerstone for algorithms focused on folding constraint satisfaction and structural refinement[111].

#### 4.4.2 Torsion angles (TA)

Protein torsion angles, also known as dihedral or rotational angles, play a pivotal role in accurately describing the local conformation of protein backbones [83]. The backbone conformation of a protein can largely be represented by two torsion angles: phi (*φ*) and psi (*ψ*), associated with each C*α* atom. The phi (*φ*) angle involves the backbone atoms C-N- C*α*-C. The psi (*ψ*) angle involves N-C*α*-C-N. The omega (*ω*) angle involves C*α*-C-N-C*α*. Due to the planarity of the peptide bond, a result of its partial double-bond character, the *ω* angle is almost always fixed at 180 (trans conformation) with rare cis cases at 0 [115]. Consequently, *φ* and *ψ* are generally sufficient to accurately describe the local shape of a protein. Therefore, if the values of the phi (*φ*) and psi (*ψ*) angles are known, the geometry of the global protein structure can be readily constructed with standard bond lengths [115]. Torsion angles *φ* and *ψ* describe the rotation between successive backbone planes, capturing the flexibility and periodicity of protein backbones. These angles typically range from 0 to 360, but are predominantly observed within specific ranges, governed by the chemical and physical properties of proteins. For instance, *φ* and *ψ* angles are clustered into distinct regions in the Ramachandran plot, reflecting steric constraints and preferred conformations. In predictive modeling, torsion angles can be represented as real-valued outputs or discrete states.Studies have shown that real-valued predictions provide more detailed insights into local backbone flexibility than discrete-state predictions. For example, Xue et al. demonstrated that real-valued predictions of torsion angles are more effective for protein structure prediction [83]. Additionally, Cheung et al. achieved 82.1 % accuracy in predicting 4-state torsion angle propensity indices (TAPI), showcasing the utility of discrete-state predictions in specific applications [83]. Tools like SPINE X and SPINE XI further combined discrete and continuous real-valued torsion angle predictions to achieve enhanced accuracy and resolution [114]. Torsion angle prediction is essential for understanding local conformational preferences and plays a critical role in protein structure prediction. Advances in this field, particularly with methods focusing on real-valued predictions, enable precise modeling of protein backbone dynamics and flexibility, which are crucial for under- standing protein folding and function.

#### 4.4.3 CN, CD, and TA Predictors

Several tools leverage evolutionary information to predict CN or TA. ANGLOR, a composite machine-learning tool, uses neural networks (NN) for phi angles and support vector machines (SVM) for psi angles. By incorporating sequence-derived features such as PSSMs, secondary structure (SS), and solvent accessibility (SA), ANGLOR achieves mean absolute errors (MAEs) of 28 and 46 for phi and psi angles, respectively, representing a significant step in applying machine learning to predict torsion angles [115].

While ANGLOR relies on traditional machine learning techniques, the introduction of deep learning in tools like Brewery and AcconPred has revolutionized the prediction of multiple structural features. For instance, Brewery [113] employs cascaded bidirectional re- current and convolutional neural networks to predict features like contact density (CD) in four states and torsion angles (TA) in 14 structural motifs. It also predicts secondary structure and RSA, leveraging evolutionary profiles from databases like UniProt20, UniRef90, and BFD. Additionally, AcconPred utilizes a multitask learning framework based on Conditional Neural Fields (CNF) to predict CN in 15 states. By modeling interdependencies among residues and nonlinear relationships, AcconPred outputs probabilistic distributions, enabling downstream structural analysis and folding constraints.

A major advancement in multitask learning is demonstrated by OPUS-TASS [116], which integrates CNN layers, bidirectional LSTM layers, and modified Transformer layers. This ensemble architecture predicts torsion angles (TA), secondary structure (SS), solvent accessibility (ASA), and side-chain dihedral angles (SDA). Innovative features such as PSP19 (rigid-body classification) and CSF3 (local backbone descriptors) further enhance its multitask learning framework. OPUS-TASS achieves state-of-the-art accuracy, outperforming earlier tools like SPOT-1D on benchmark datasets.

The transition to deep learning has further introduced advanced multitask models such as IGPRED-MultiTask [83], which combines convolutional neural networks (CNNs), graph convolutional networks (GCNs), and bidirectional LSTM layers. This model predicts torsion angles (phi and psi) as real-valued outputs, while simultaneously predicting secondary structure and solvent accessibility. Optimized through Bayesian techniques and trained using sequence and structural profiles, IGPRED-MultiTask showcases the power of multitask learning in handling complex sequence-to-structure relationships.

With the rise of protein language models (PLMs), tools like SPOT-1D-LM and NetSurfP-3.0 also predict CN and backbone angles, achieving high accuracy. NetSurfP-3.0 predicts CN and torsion angles (phi and psi) while bypassing traditional multiple sequence alignment. This approach significantly reduces computational overhead while maintaining high accuracy, providing valuable insights into protein structure prediction.

## 5 Evaluation metrics for model performance

The DOME (Data, Optimization, Model, Evaluation) recommendations emphasize transparency and reproducibility in machine learning-based research. These guidelines highlight the importance of detailed reporting on data sources, optimization strategies, model architectures, and evaluation metrics to improve reliability. In the context of protein prediction models, adopting the DOME framework can enhance robustness and facilitate reproducibility in future research[117].

Following the DOME recommendations, Model validation is a critical step to guarantee that neural networks deliver dependable outcomes in real-world applications. It plays a key role in preventing data overfitting, enabling an unbiased assessment of model performance, and enhancing both the credibility and transparency of the model. In bioinformatics, data often exhibit significant complexity and heterogeneity. By employing cross-validation and external validation, researchers can ensure the model’s robustness across diverse datasets and conditions. This is particularly crucial given the vast amounts of data produced by high-throughput sequencing technologies, emphasizing the need to confirm the model’s performance on unseen data [117].

Accuracy measures the proportion of correct predictions (both true positives and true negatives) out of the total number of cases evaluated. It is mathematically expressed as:

$$\mathrm{Accuracy}=\frac{\mathrm{TP}+\mathrm{TN}}{\mathrm{TP}+\mathrm{TN}+\mathrm{FP}+\mathrm{FN}}\;$$

Here, TP refers to true positives, TN to true negatives, FP to false positives, and FN to false negatives.

However, accuracy alone can be misleading in imbalanced datasets, where one class is significantly underrepresented. In such cases, a model may achieve high accuracy simply by predicting the majority class, failing to properly recognize the minority class.

Balanced Accuracy addresses this issue by averaging recall values for each class, ensuring that both majority and minority classes contribute equally. It is defined as:

$$\mathrm{Balanced accuracy}=\left(\mathrm{Sensitivity}+\mathrm{Specificity}\right)/2$$

Where Sensitivity is the true positive rate and the Specificity is the true negative rate.This metric is particularly useful when dealing with highly skewed datasets, as it prevents overestimation of model performance in cases where one class dominates.

Precision represents the proportion of true positive predictions among all the samples the model predicted as positive. Recall, on the other hand, indicates the proportion of true positive samples that the model correctly identified as positive. These metrics are particularly critical for imbalanced datasets. They are defined as follows:

$$\mathrm{Precision}=\frac{\mathrm{TP}}{\mathrm{TP}+\mathrm{FP}}$$

$$\mathrm{Recall}=\frac{\mathrm{TP}}{\mathrm{TP}+\mathrm{FN}}$$

The F1 score combines precision and recall into a single metric, representing their harmonic mean. It provides a balanced measure of both metrics and is defined as:

$$F1\mathrm{Score}=2\times \frac{\mathrm{Precision}\times \mathrm{Recall}}{\mathrm{Precision}+\mathrm{Recall}}$$

The Area Under the Curve - Receiver Operating Characteristic (AUC-ROC) is a performance metric for classification tasks evaluated at various threshold settings. The ROC curve is a graphical representation of the true positive rate (TPR) plotted against the false positive rate (FPR). The AUC measures the model’s ability to distinguish between classes, with higher values indicating better class separability.

$$\mathrm{AUC}=\int_{0}^{1}\mathrm{TPR}({\mathrm{FPR}}^{-1}(t))\mathrm{dt}$$

TPR and FPR are the true positive rate and false positive rate, respectively. t is the decision threshold. ${\mathrm{FPR}}^{-1}$ represents the inverse of the false positive rate function.

The Pearson correlation coefficient measures the linear correlation between two variables, indicating the strength and direction of the linear relationship between predicted and actual values. It is mathematically represented as:

$$\mathrm{PCC}=\frac{\sum_{i=1}^{n}\left(\mathrm{xi}-\;\overset{®}{x})(\mathrm{yi}-\;\overset{®}{y}\right)}{\sqrt{\sum_{i=1}^{n}{\left(x_{i}-\overset{®}{x}\right)}^{2}}\sum_{i=1}^{n}{{(y}_{i}-\overset{®}{y})}^{2}}$$

Where xi and yi are the predicted and actual values $\overset{®}{x}$ and $\overset{®}{y}$ are the means of the predicted and actual values, respectively.

Precision Score (APS) is a robust metric for evaluating model performance on imbalanced datasets. It assesses the model’ stability to differentiate between classes cross all threshold levels. APS is particularly beneficial in scenarios with significant class imbalance, where the primary focus is on the positive class, such as in intrinsically disordered region (IDR) prediction. It is calculated as:

$$\mathrm{APS}=\frac{\sum_{K=1}^{N}\left({\mathrm{Sensitivity}}_{K}\;-{\;\mathrm{Specificity}}_{K}\right)}{N}$$

${\mathrm{Sensitivity}}_{K}$and ${\mathrm{Specificity}}_{K}$ are the sensitivity (recall) and specificity at thresholdK,respectively. N is the total number of thresholds, and K is the threshold index.

Importance of Choosing the Right Metrics for Imbalanced Datasets In imbalanced datasets, metrics like accuracy can be inappropriate, as a model may predict the majority class overwhelmingly and still achieve high accuracy despite poor predictive performance on the minority class. Instead, balanced accuracy, precision-recall, F1 score, and APS provide a more reliable assessment of a model’s performance in such cases by emphasizing minority class detection and penalizing false positives or false negatives accordingly.

These metrics collectively provide a comprehensive framework for evaluating the performance of predictive models, ensuring their applicability and reliability across diverse bioinformatics challenges.

## 6 Trends in 1D protein structure prediction

### 6.1 The AlphaFold revolution and its impact

AlphaFold [118], [119] by DeepMind achieved what was once deemed impossible: a ground- breaking level of accuracy in protein structure prediction. The initial version demonstrated the potential of neural networks to extract protein-specific potentials directly from sequence data, leveraging deep convolutional architectures to generate high-quality distance distributions and backbone torsion angles. However, the release of AlphaFold2 set new standards by integrating evolutionary, physical, and geometric constraints into an innovative neural network architecture, fundamentally reshaping the field [119].

AlphaFold2 introduced the Evoformer and Structure Module—two neural networks that transformed how sequence-based multiple sequence alignments (MSAs) and pairwise structural templates are integrated [119], [120]. The Evoformer, composed of 48 blocks, enables communication between MSA and pair representations through a combination of attention- based and non-attention-based mechanisms. It employs advanced operations, including triangle multiplicative updates and self-attention, to extract spatial and evolutionary relationships between residues. Leveraging these relationships, the Structure Module then generates 3D structural models, positioning side chains and residues accurately based on backbone frames and other geometric constraints.

Despite its remarkable performance, AlphaFold2 has notable limitations. It underperforms in predicting intrinsically disordered regions (IDRs), accurately modeling loop conformations, and accounting for large conformational variations between holo and apo states. Moreover, AlphaFold2 relies heavily on evolutionary information, requiring a minimum of 30 effective homologous sequences to achieve accurate predictions [75]. This dependency leaves proteins with sparse homologous sequences—particularly those without robust evolutionary data—beyond its predictive capability. Generating MSAs from vast protein databases can also be computationally intensive, underscoring the need for structure prediction methods that do not rely on extensive evolutionary information [26].

A key factor in AlphaFold’s success is its ability to bypass intermediate tasks such as secondary structure prediction (PSSP), fold prediction, and structural feature extraction, predicting tertiary structures directly from primary sequences [26]. Although AlphaFold primarily focuses on 3D structure prediction, it integrates 1D structural annotations such as secondary structure, torsion angles, and solvent accessibility as intermediate representations to refine its predictions. These structural features contribute to the network’s understanding of sequence-structure relationships, allowing AlphaFold to enhance its accuracy even when dealing with challenging protein folds. However, for proteins with limited evolutionary information, complementary strategies—especially those predicting backbone structures and 1D structural properties—remain crucial.

Building on AlphaFold2’s foundation, the newly introduced AlphaFold3 [121] offers an enhanced capacity to predict not only the structure of a single protein but also interactions between multiple molecules. This advancement could significantly benefit 1D sequence prediction tasks, such as protein aggregation predictions [122], [123], by providing a more comprehensive view of inter-sequence or inter-molecular interactions. As AlphaFold3 continues to evolve, it may further streamline how 1D and 3D protein structure data are integrated, especially for complex systems where multiple sequences or molecular partners interact.

### 6.2 The rise of protein language models (PLMs)

Protein Language Models (PLMs) represent a paradigm shift in 1D protein structure prediction, taking inspiration from natural language processing (NLP) to interpret protein sequences as "sentences" and amino acids as "words [124]." Unlike AlphaFold, which relies heavily on multiple sequence alignments (MSAs) and structural templates to achieve high accuracy, PLMs like ProtT5 and ESM work directly on raw protein sequences. This ability to learn evolutionary and functional patterns from large-scale datasets without requiring MSAs highlights a fundamental difference between the two approaches [125].

PLMs such as SeqVec and ProtTrans leverage advanced architectures, including bidirectional LSTMs and Transformers, to encode the underlying biological properties of amino acid sequences. SeqVec, for instance, adapts the ELMo model from NLP, creating continuous embeddings that improve predictions across various 1D structural tasks without the computationally intensive step of generating MSAs. Similarly, ProtTrans expands on this concept, using Transformers to generate embeddings that enhance predictions for tasks such as three-state secondary structure classification and solvent accessibility estimation.

One of the key advantages of PLMs lies in their computational efficiency and accessibility. By avoiding the need for sequence profiles like PSSMs or HMMs, PLMs significantly reduce the time and resources required for 1D PSP workflows. This makes them particularly well-suited for applications involving orphan proteins or metagenomic sequences, where homologous evolutionary information is often unavailable. However, while PLMs have advanced the field considerably, they currently act as a complement rather than a replacement for traditional evolutionary profile-based methods. For some tasks, particularly those re- quiring high-resolution accuracy, PLMs still lag behind approaches like AlphaFold, which effectively integrate MSAs and structural templates.

Despite their differences, PLMs and AlphaFold share the broader goal of advancing protein structure prediction. While AlphaFold excels in predicting high-resolution tertiary structures, PLMs offer flexibility and scalability, enabling predictions in cases where MSAs are impractical or unavailable [3].However, PLMs and AlphaFold differ fundamentally in their reliance on data and computational design. PLMs, such as ESM-2 and ProtT5, predict structural properties directly from single sequences, making them faster and applicable to orphan proteins. In contrast, AlphaFold depends on MSAs and structural templates, which improve accuracy but require intensive sequence searches and higher computational costs. While AlphaFold is optimized for atomic-level 3D structures, PLMs are more effective for predicting sequence-based structural features such as secondary structure and disorder regions. By removing evolutionary constraints, PLMs offer a complementary strategy to AlphaFold, particularly for rapid protein annotation and low-homology sequences. Together, these methods illustrate the diversity of strategies emerging in computational biology, with each playing a distinct role in addressing the multifaceted challenges of protein structure prediction.

### 6.3 Challenges and future directions

Despite remarkable advancements in 1D protein structure prediction (PSP), several challenges remain that hinder achieving comprehensive accuracy and applicability across diverse protein datasets. These challenges arise from limitations in data availability, model generalization, and computational constraints.

A primary concern is the reliance on high-quality data, particularly for proteins lacking extensive evolutionary information or for which only low-resolution experimental structures are available. Methods ranging from Protein Language Models (PLMs) to more traditional approaches often struggle to maintain accuracy for orphan proteins and metagenomic sequences. Although PLMs reduce the need for multiple sequence alignments (MSAs), their generalization is constrained by the scarcity of annotated datasets covering underrepresented protein families. This situation is further compounded by the mismatch between the relatively small number of experimentally resolved 3D protein structures and the massive volume of sequence data.

Another significant challenge is the development of accurate predictors for small functional regions, such as intrinsically disordered regions (IDRs), linkers, and binding sites. These regions are often context-dependent, transient, or exhibit minimal sequence homology, making it difficult to compile large, representative datasets. Moreover, many of their functional roles rely on dynamic conformations or specific interaction partners—factors not readily captured by static sequence data alone. Consequently, building robust predictors necessitates not only sophisticated models capable of handling these intricacies but also more comprehensive datasets that incorporate experimental evidence, sequence diversity, and, wherever possible, structural, or biophysical information.

Another challenge lies in the interpretability of deep learning models. Both PLMs and other machine learning-based predictors are often described as "black-box" systems, offering little insight into how predictions are made. While deep learning models are often seen as 'black boxes,' interpretability can still be achieved using various techniques designed to explain model decisions. However, even inherently interpretable models, such as decision trees, can become impractical when handling thousands of variables due to complexity and feature interactions. Therefore, model selection should balance predictive performance and interpretability, ensuring practicality for real-world applications. This lack of interpretability raises concerns about reliability and hinders their adoption in critical applications, such as drug discovery and personalized medicine. Efforts are underway to develop explainable AI frameworks that can provide rationales for predictions while retaining the accuracy and scalability of existing models.

Furthermore, the computational demand of large-scale PLMs poses a significant barrier. Models such as ProtTrans and ESM rely on extensive datasets and massive computational re- sources for pre-training and fine-tuning. These requirements limit accessibility to researchers with restricted computational infrastructure, hindering broader adoption. Addressing this issue requires the development of more efficient architectures and training strategies, such as smaller yet effective PLMs optimized for specific tasks without compromising performance. Looking to the future, the integration of multimodal approaches offers a promising direction. Combining sequence-based representations from PLMs with structural and physicochemical data could enhance prediction accuracy and expand the applicability of 1D PSP methods to novel protein families and dynamic systems. Additionally, advancements in self- supervised learning and pre-training strategies, such as contrastive learning, may further improve the ability of models to capture complex relationships within protein data.

Moreover, hybrid models that synergize traditional sequence-based methods with PLMs can address some of the current limitations. For instance, leveraging evolutionary profiles alongside PLM embeddings could provide complementary insights, especially for tasks re- quiring fine-grained structural predictions. This collaborative approach may bridge the gap between models optimized for raw sequence data and those that depend on evolutionary information.

Finally, fostering open-access datasets and standardized benchmarks for evaluating 1D PSP methods will play a critical role in advancing the field. Initiatives to curate diverse, high- quality datasets encompassing underrepresented protein families will empower researchers to develop and validate more robust models. Additionally, adopting unified metrics for model evaluation will facilitate fair comparisons and accelerate innovation across the field.

In summary, while significant strides have been made in 1D PSP, addressing the challenges of data scarcity, model interpretability, and computational efficiency will be pivotal for future advancements. By embracing multimodal learning, hybrid approaches, and collaborative data-sharing efforts, the field is well-positioned to tackle these challenges and unlock the full potential of protein structure prediction.

## 7 Conclusion

This review highlights the significant progress made in 1D protein structure prediction (PSP), focusing on new methods and the role of deep learning. It discusses how Protein Language Models (PLMs) have become important tools in predicting protein features, especially for sequences without evolutionary data. The paper also compares the contributions of AlphaFold and PLMs, showing how each approach helps improve our understanding of protein structure.

Key points include the efficiency of PLMs, their ability to work with raw sequence data, and the growing move toward methods that do not rely on alignments. The review also discusses important trends, such as using multitask learning and large datasets to improve predictions. However, challenges like the need for high-quality datasets, easier-to-understand models, and better access to computational tools still remain.

By analyzing current methods and identifying areas for growth, this review provides guidance for future work in the field. Improving hybrid methods, combining data from different sources, and making advanced tools more accessible will help further improve protein structure predictions. These advances will support research in drug discovery, protein function studies, and synthetic biology.

## CRediT authorship contribution statement

**Alanazi Wafa J:** Writing – review & editing, Writing – original draft. **Meng Di:** Writing – review & editing. **Pollastri Gianluca:** Writing – review & editing.

## Declaration of Competing Interest

We declare that we do not have any conflict of interest.
